# Fuzheng Huayu formula ameliorates chronic cholestatic liver injury by upregulating PPARa in mice

**DOI:** 10.1186/s13020-026-01368-2

**Published:** 2026-03-18

**Authors:** Zheng Zhang, En-qi Tang, Chun-hui Li, Bi-bi Wang, Yue Liang, Jin-xin Lv, Gao-feng Chen, Wei Liu, Yong-ping Mu, Ping Liu, Jia-mei Chen

**Affiliations:** 1 Institute of Liver Diseases, Key Laboratory of Liver and Kidney Diseases (Ministry of Education), Shuguang Hospital affiliated to Shanghai University of Traditional Chinese Medicine, 528 Zhangheng Road, Shanghai, 201203 China; 2 Institute of Interdisciplinary Medicine, Shanghai University of Traditional Chinese Medicine, Shanghai, 201203 China

**Keywords:** Fuzheng Huayu, PPARα, Bile acids, Inflammation, Biliary fibrosis

## Abstract

**Background:**

Fuzheng Huayu formula (FZHY) has been extensively applied in clinical for liver fibrosis treatment in China, its therapeutic potential in cholestatic liver injury remains underexplored.

**Objective:**

To evaluate the protective effects and underlying mechanisms of FZHY against chronic cholestatic liver injury.

**Methods:**

The therapeutic effect of FZHY was initially validated in a 3,5-diethoxycarbonyl-1,4-dihydrocollidine (DDC)-induced murine model of chronic cholestasis. Subsequent mechanistic investigations were conducted through comparative analyses in peroxisome proliferator-activated receptor α gene knockout (*Pparα*^−/−^) mice subjected to DDC challenge.

**Results:**

FZHY significantly ameliorated chronic cholestatic liver injury phenotypes in DDC-induced mice, as evidenced by bile acids (BAs) accumulation, inflammation, ductular reaction and biliary fibrosis was remarkably reduced after treatment with FZHY. Transcriptome sequencing analysis revealed that the effect of FZHY on chronic cholestatic liver injury was closely associated with activating PPAR signaling pathway and suppressing nuclear factor kappa-B (NF-κB) signaling. Further research found FZHY did not only enhance the total hepatic content of PPARα protein, but also increased its nuclear to cytoplasmic ratio that was reduced by DDC inducing. Additionally, FZHY suppressed hepatic phosphorylation of IκBα and NF-κB. The therapeutic effect of FZHY in treating DDC-induced mice with chronic cholestatic liver injury is similar to that of fenofibrate, a PPARα agonist. Crucially, genetic ablation of *Pparα* substantially abrogated the hepatoprotective and anti-fibrotic effects of FZHY in DDC-induced mice.

**Conclusions:**

The present study underscores FZHY regulated BAs metabolism and alleviated hepatic inflammation and fibrosis by upregulating PPARa in DDC-induced mice. Our study provides novel insights that FZHY might be a promising drug for chronic cholestatic liver injury.

**Graphical Abstract:**

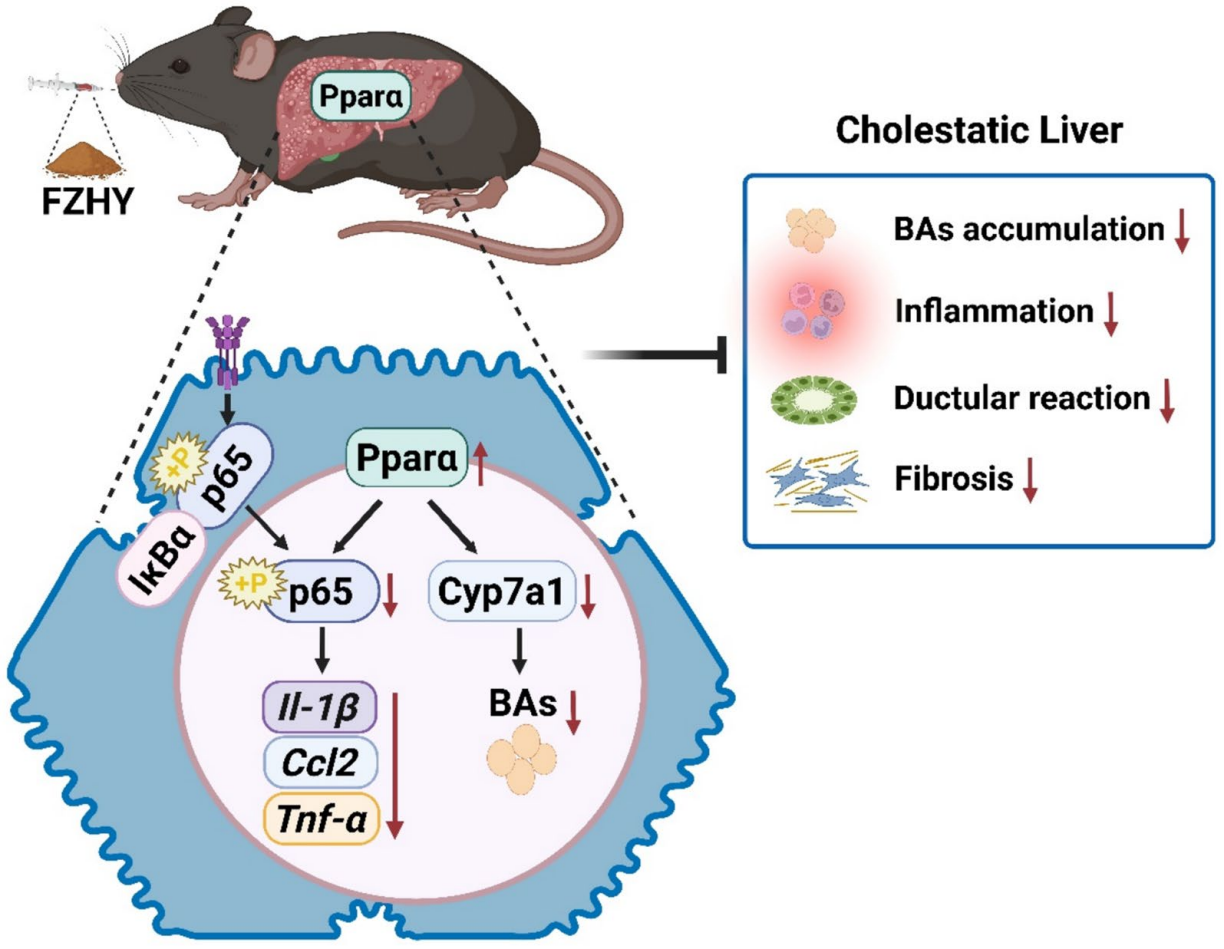

**Supplementary Information:**

The online version contains supplementary material available at 10.1186/s13020-026-01368-2.

## Introduction

Cholestatic liver diseases arise from progressive destruction of the biliary architecture, pathological bile acids (BAs) accrual, and self-amplifying inflammatory cascades. This pathophysiological triad drives progressive epithelial injury through cholangiocyte-hepatocyte crosstalk disruption, culminating in parenchymal degeneration. Primary sclerosing cholangitis (PSC) and primary biliary cholangitis (PBC) are the two most common chronic cholestatic liver diseases. Unless successfully treated, cholestasis leads to biliary fibrosis, cirrhosis, and ultimately end-stage liver disease [1, 2]. Ursodeoxycholic acid therapy significantly improves outcomes and prolongs transplant-free survival in PBC patients [3], but with problems such as incomplete response, and heavy economic burden [4, 5]. Liver transplantation remains the only life-extending treatment for many patients with end-stage cholestatic liver disease. Therefore, exploring effective therapeutic approaches for cholestatic liver injury holds significant clinical importance and meets critical societal needs.

Peroxisome proliferator-activated receptors (PPARs), including PPARα, PPARγ, and PPARβ/δ, belong to the nuclear hormone receptor superfamily and are involved in regulating energy metabolism, cell development, and cell differentiation [6]. PPARα plays a major role in the liver by regulating the transcription of genes associated with β-oxidation, gluconeogenesis, and cholesterol metabolism [7]. PPARα agonists have been reported to regulate BAs homeostasis by inhibiting BAs synthesis and interacting with FXR signals. Recent studies have shown that activating PPARs can enhance the response to UDCA inpatients with cholestatic liver disease [8, 9]. Furthermore, PPARα can inhibit the expression of pro-inflammatory genes in a ligand-dependent manner. Owing to its function in BAs metabolism, anti-inflammation, and anti-oxidation, PPARα has emerged as a potential target for controlling the progression of cholestasis [10].

Fuzheng Huayu formula (FZHY) is widely used in clinical to treat liver fibrosis and cirrhosis caused by various chronic liver diseases in China. It demonstrates efficacy in improving serum biochemical parameters, liver histopathology, and serological markers of liver fibrosis, as well as reducing liver stiffness, portal hypertension, hepatocellular carcinoma incidence, and mortality rates in cirrhotic patients [11]. However, studies on FZHY for cholestatic liver injury remain limited.

In this study, we investigated the effects and mechanisms of FZHY in 3,5-diethoxycarbonyl-1,4-dihydrocollidine (DDC)-induced chronic cholestatic liver injury in mice. Additionally, *Pparα* gene knockout mice were used to elucidate the mechanism of FZHY. We found that FZHY significantly ameliorates BAs accumulation, inflammation, ductular reaction and biliary fibrosis induced by DDC in mice is depended on PPARα.

## Materials and methods

### Materials

FZHY (No., 180206) was provided by Shanghai Huanghai Pharmaceutical Co., Ltd. (Shanghai, China). Obeticholic acid (OCA) (No., MB6084), a farnesoid X receptor agonist, was purchased from Dalian Meilun Biotechnology Co., Ltd. (Dalian, China). Fenofibrate (FF, No., F6020) was purchased from Merck Millipore (Burlington, MA, USA). The DDC diet was formulated by supplementing standard chow with 0.1% (w/w) DDC (No., M2011, Fanbo Biotechnology Co., Ltd., Jiangsu, China).

### Preparation of animal models and drug administration

Male wild-type (WT) C57BL/6 mice (8 weeks old, body weight 22 ± 2 g) were obtained from Beijing Vital River Laboratory Animal Technology. *Pparα*^−/−^ mice on a C57BL/6 background were donated by Professor Houkai Li’s laboratory (Shanghai University of Traditional Chinese Medicine). All animals were housed within the Animal Experiment Center of Shanghai University of Traditional Chinese Medicine under controlled conditions: temperature maintained at 22–24 °C, humidity at 30–60%, and a 12-h light/dark cycle. Mice had free access to designated food and water ad libitum.

DDC-induced mice: Male WT C57BL/6 mice were randomly assigned into either a control group (*n* = 8) or model groups (*n* = 24). Control mice received standard chow ad libitum, while model group mice were fed the 0.1% (w/w) DDC-supplemented diet for 7 weeks. Beginning at week 5 of DDC feeding, surviving mice in the model group were re-randomized into three treatment subgroups (*n* = 8/group): treatment with the 0.3% CMC-Na (vehicle) as the model group, or FZHY or 10 mg/kg OCA (the positive control) by gavage daily for 3 weeks. Based on previously established protocols, the mouse-equivalent dose of FZHY, calculated from the clinical adult dose via body surface area conversion, has been shown to effectively ameliorate carbon tetrachloride (CCl₄)-induced liver fibrosis [12] and *Mdr2*^−/−^ spontaneous chronic cholestasis in mice [13]. In accordance with these evidences, FZHY was administered orally at a daily dose of 4 mg/kg in the present study. At the end of week 7, all mice were anesthetized by intraperitoneal injection of 3% pentobarbital sodium. Blood and liver tissues were subsequently collected for subsequent experiments.

DDC-induced models in WT and *Pparα*^−/−^ mice: WT and *Pparα*^−/−^ mice (age- and gender-matched) were randomized into seven experimental groups (*n* = 8/group). Mice received either standard chow or a 0.1% (w/w) DDC-supplemented diet ad libitum for 7 weeks. Beginning at week 5, the following treatments were administered via daily oral gavage for 3 weeks: vehicle (0.3% CMC-Na) to select DDC-fed WT and *Pparα*^−/−^ mice; FZHY (4 mg/kg) to select DDC-fed WT and *Pparα*^−/−^ mice; fenofibrate (FF, 100 mg/kg) to DDC-fed WT mice (positive control). At the end of week 7, all mice were anesthetized by intraperitoneal injection of 3% pentobarbital sodium, followed by blood and tissue collection for subsequent analyses.

### RNA sequencing and bioinformatic analysis

Total RNA was extracted and used for cDNA library construction. Libraries were sequenced on an Illumina NovaSeq™ 6000 platform (Illumina, San Diego, CA, USA) to generate 150-bp paired-end reads. Transcript abundance was quantified as FPKM (Fragments Per Kilobase of transcript per Million mapped reads) using Cufflinks. Raw gene counts were obtained with HTSeq-count. Differential expression analysis was performed using DESeq2 (v3.2.0) in R, with significantly differentially expressed genes (DEGs) defined by a nominal *P*-value < 0.05 and |log₂ fold-change|> 0.585 (equivalent to fold-change > 1.5 or < 0.67).

Hierarchical clustering of DEGs was performed to visualize expression patterns across experimental groups. Gene ontology (GO) and kyoto encyclopedia of genes and genomes (KEGG) pathway enrichment analyses were conducted in R using the hypergeometric distribution test. Gene set enrichment analysis (GSEA) was implemented using the GSEA software (Broad Institute). For GO/KEGG analyses, gene sets corresponding to enriched terms were defined, and significance was determined by permutation testing (gene set permutation type) with normalized enrichment scores (NES). A false discovery rate (FDR) < 0.05 was set as the significance threshold for enriched terms. All transcriptome sequencing and bioinformatic analyses were performed by OE Biotech Co., Ltd. (Shanghai, China).

### Hydroxyproline (Hyp) content measurement

Hepatic Hyp content was quantified using a commercial Hyp testing kit (alkaline hydrolysis method; A030-2-1, Nanjing Jiancheng Institute of Biological Engineering Institute, Nanjing, China) according to the manufacturer’s protocol.

### Histopathological and immunohistochemical analysis

Liver specimens were fixed in 10% neutral-buffered formalin, dehydrated through an automated graded ethanol series using a vacuum tissue processor, paraffin-embedded, and sectioned at 4 μm thickness. Sections were stained with hematoxylin and eosin (H&E) to assess hepatic histoarchitecture and injury, sirius red (SR) to evaluate collagen deposition and fibrosis severity. For immunohistochemical analysis, sections were incubated with primary antibodies (Supplementary Table 1) according to manufacturers’ protocols. All stained sections were digitized using a KF-PRO-400-HI whole-slide scanner (Konfoong Bioinformation Technology Co., Ltd., Ningbo, China).

### Western blot analysis

Total protein was extracted from liver tissues using RIPA lysis buffer (containing protease inhibitors). Protein concentrations were quantified using the bicinchoninic acid assay. Equal amounts of protein (30 μg per lane) were separated by SDS-PAGE and electrophoretically transferred to polyvinylidene difluoride membranes. Membranes were blocked, then incubated overnight at 4 °C with primary antibodies (Supplementary Table 1). After three 10-min washes with TBST, membranes were incubated with horseradish peroxidase (HRP)-conjugated secondary antibodies for 1 h at room temperature. Immunoreactive bands were detected using enhanced chemiluminescence reagents (Tanon 5200, Shanghai, China) and imaged with a chemiluminescence documentation system.

### Quantitative reverse transcript polymerase chain reaction (qRT-PCR)

Total RNA was isolated from liver tissues using the Total RNA Extraction Kit. RNA was reverse-transcribed into cDNA using the Reverse Transcription Kit according to the manufacturer’s protocol. qRT-PCR was performed on an Applied Biosystems™ ViiA™ 7 Real-Time PCR System (Foster City, CA, USA) using Power SYBR^®^ Green Master Mix. Relative mRNA expression levels were calculated using the comparative ^ΔΔ^Ct method normalized to *Gapdh*. The primers sequences used in this study were presented in Supplementary Table 2.

### Determination of hepatic bile acids content

Approximately 50 mg of liver tissue was precisely weighed and placed into a 1.5 mL microcentrifuge tube. 1 mL of an internal standard working solution containing mixed isotope-labeled internal standards at a concentration of 10 ng/mL was added to the tube, followed by the addition of two steel beads. The mixture was homogenized for 2 min. The homogenate was centrifuged at 15,000 rpm (revolutions per minute) for 10 min at 4 °C. Following centrifugation, 800 µL of the supernatant was carefully transferred to a new 1.5 mL microcentrifuge tube. The supernatant was evaporated to dryness under a gentle nitrogen stream using a nitrogen evaporator. The dried residue was reconstituted by adding 160 µL of 70% methanol and vortex-mixing for 1 min. The reconstituted solution was centrifuged again at 15,000 rpm for 10 min at 4 °C. Finally, 100 µL of the resulting supernatant was transferred to an autosampler vial insert for subsequent UPLC-MS analysis.

### Statistical analysis

Statistical analyses were performed using GraphPad Prism version 8.0 (GraphPad Software, San Diego, CA, USA). One-way ANOVA analysis is used for the comparative analysis of multiple groups, and Student’s *t*-test is used for the statistical comparison between two groups. Statistical significance was defined as *P* < 0.05.

## Results

### FZHY ameliorated ductular reaction and biliary fibrosis in DDC-induced chronic cholestatic liver injury mice

**Fig. 1:**
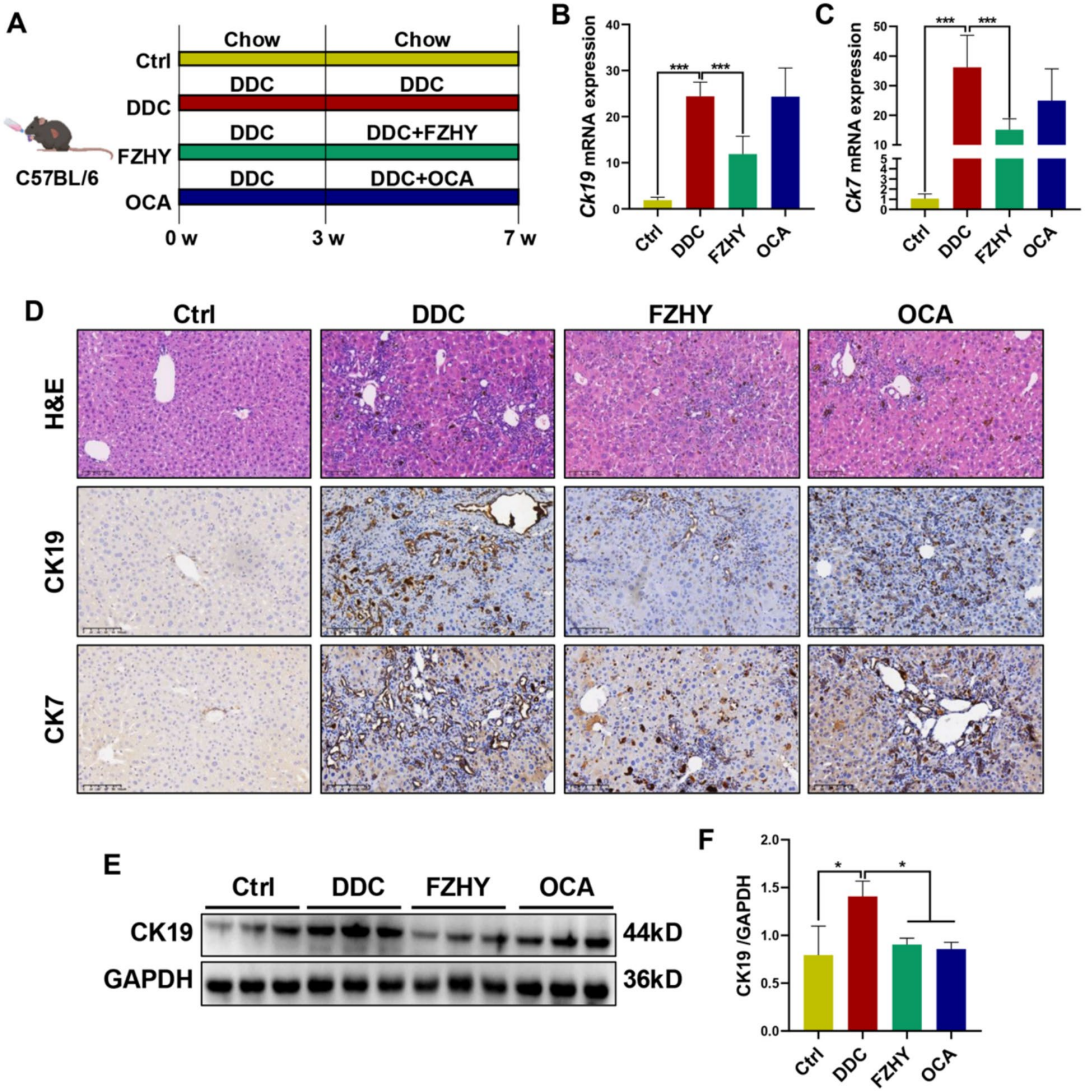
FZHY ameliorated ductular reaction in DDC-induced mice. **A** Schematic diagram illustrating the experimental design of DDC-induced mice treated with FZHY or OCA. **B, C** *Ck19* and *Ck7* expressions were determined by qRT-PCR. **D** Liver sections were stained with H&E (scale bar = 100 µm), CK19 (scale bar = 100 µm) and CK7 (scale bar = 100 µm). Representative images are shown. **E, F** Immunoblotting and quantification of CK19. *P < 0.05; **P < 0.01; ***P < 0.001

To investigate the therapeutic potential of FZHY in chronic cholestatic liver injury, we employed a well-established murine model of diet-induced cholestasis through 7-week administration of a DDC-enriched diet. OCA was served as the positive control (Fig. 1A). Histopathological analysis via H&E staining revealed characteristic DDC-induced hepatic pathology, including periportal hepatocyte degeneration/necrosis and intrahepatic bile duct hyperplasia. Notably, both FZHY and OCA treatment groups exhibited marked reductions in these histopathological aberrations (Fig. 1D). Consistently, FZHY treatment significantly attenuated hepatic expression of cholangiocyte markers CK19 and CK7 (*P* < 0.05, *P* < 0.01), as evidenced by qRT-PCR (Fig. 1B, C), immunohistochemical saining (Fig. 1D) and western blot (Fig. 1E, F).

**Fig. 2:**
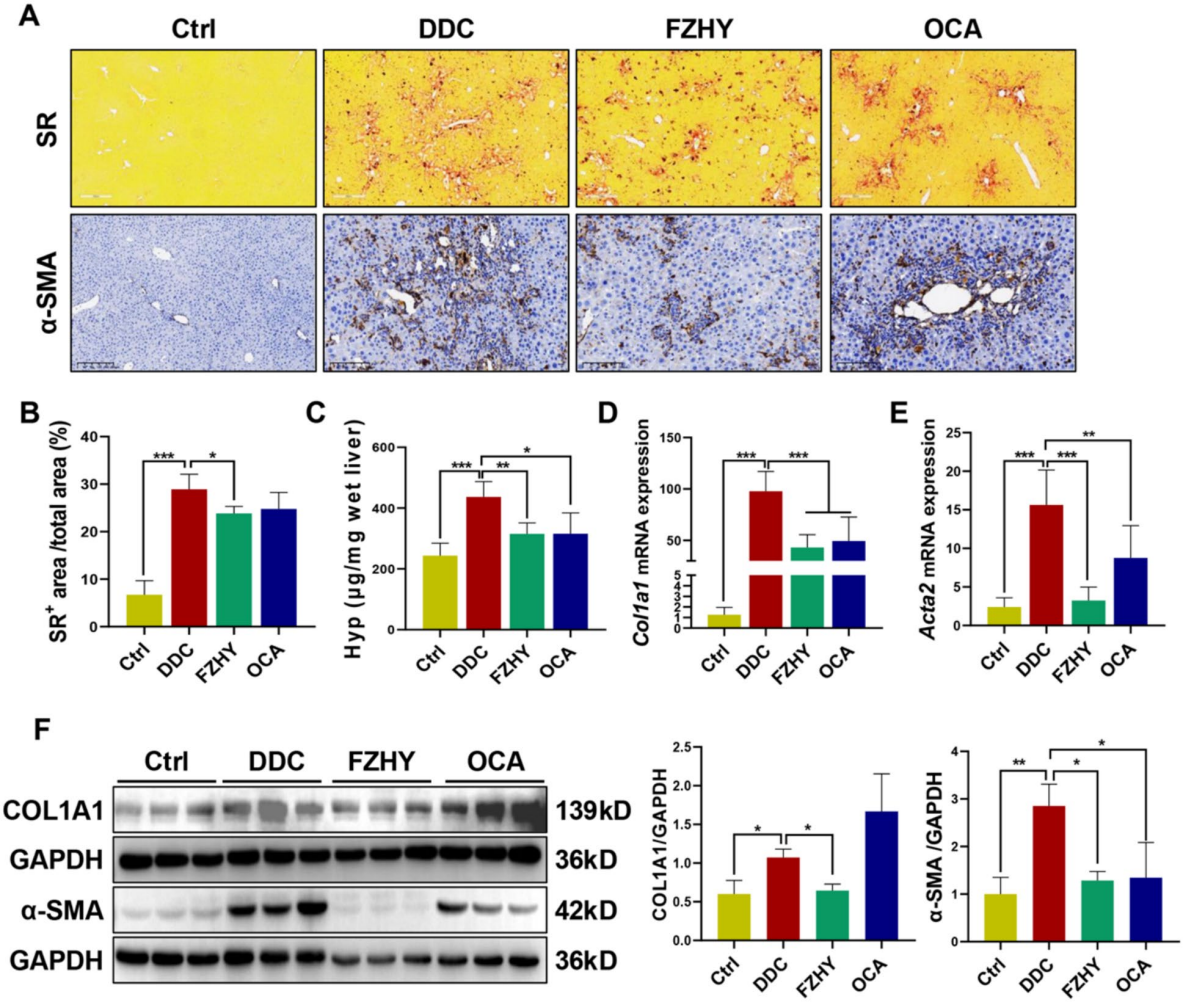
FZHY ameliorated biliary fibrosis in DDC-induced mice. **A** Liver sections were stained with SR staining (scale bar = 200 µm) and α-SMA (scale bar = 100 µm). **B** Collagen morphometry (%) of SR^+^ area. **C** Hepatic collagen content was biochemically determined by Hyp analysis. **D, E** *Col1a1* and *Acta2* expression was determined by qRT-PCR. **F** Immunoblotting and quantification of COL1A1 and α-SMA. *P < 0.05; **P < 0.01; ***P < 0.001

Histochemical analyses revealed that chronic DDC exposure induced substantial collagen deposition and periportal fibrogenesis, as evidenced by SR staining and immunohistochemical saining of α-SMA. Notably, FZHY intervention demonstrated marked attenuation of DDC-induced biliary fibrosis (Fig. 2A). Consistent with histopathological findings, the percentage of SR^+^ area (*P* < 0.05), hepatic Hyp content (*P* < 0.01), as well as hepatic expression of COL1A1 and a-SMA (*P* < 0.05, *P* < 0.01, *P* < 0.001) showed by western blot and qRT-PCR results, demonstrated significant reductions in FZHY-treated mice compared to DDC-induced mice (Fig. 2B-F).

### FZHY improved BAs metabolism by regulating PPARα signaling pathway

**Fig. 3:**
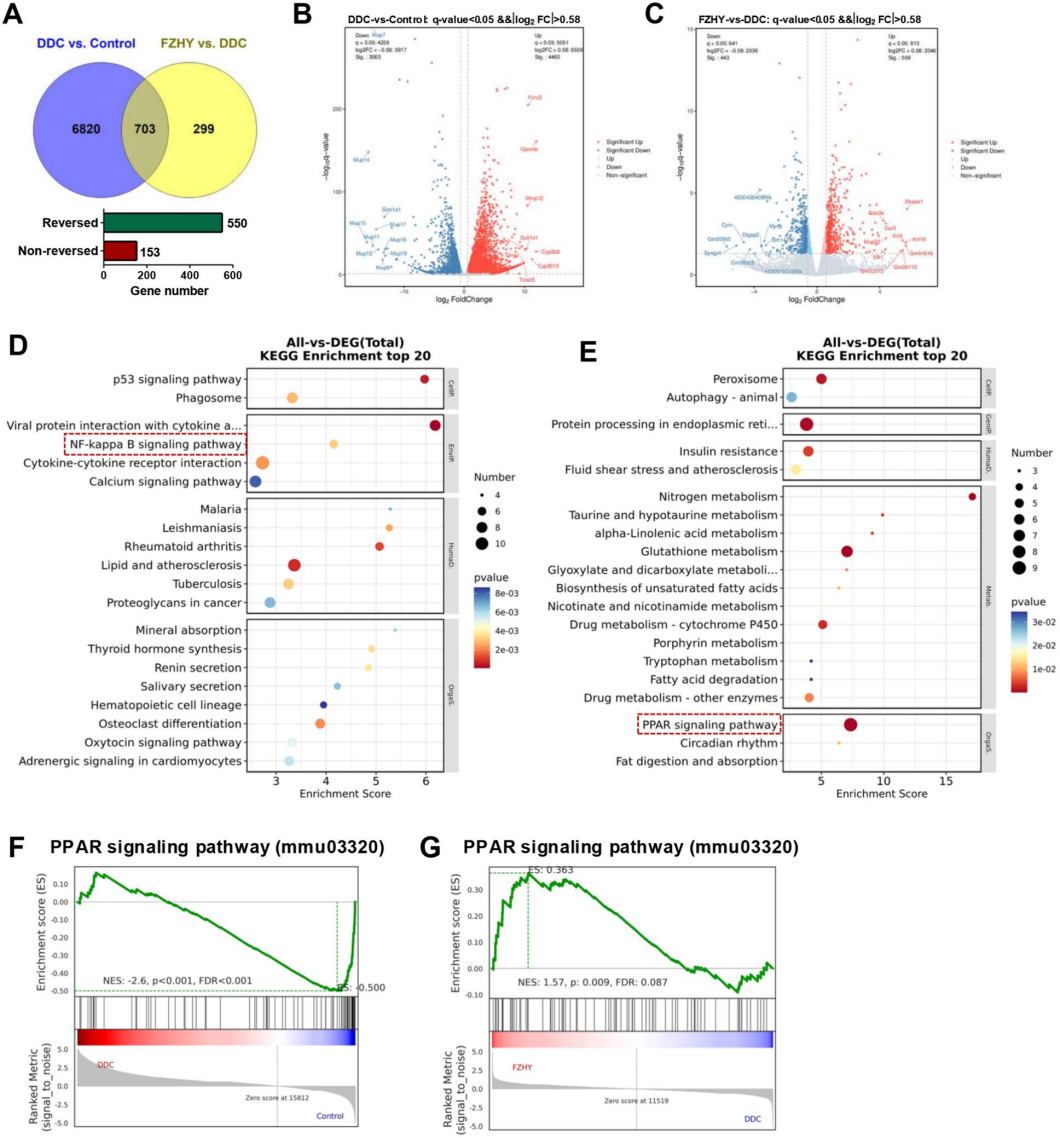
FZHY activated PPARα signaling in DDC-induced liver. **A** Venn diagram of significantly regulated genes by DDC or by FZHY as shown by transcriptomics (*n* = 3 per group). **B, C** The volcanic map of DEGs between DDC vs. Control (**B**) and DDC + FZHY vs. DDC (**C**). **D** Top 20 pathways enriched with KEGG enrichment analysis based on DDC-upregulated/FZHY-downregulated genes. **E** Top 20 pathways enriched with KEGG enrichment analysis based on DDC-downregulated/FZHY-upregulated genes. **F** GSEA analysis of PPAR signaling pathway in DDC-induced livers. **G** GSEA analysis of PPAR signaling pathway in FZHY-treated livers

To delineate the mechanistic basis of FZHY efficacy, hepatic transcriptomic profiling was conducted via RNA sequencing (RNA-seq). Comparative analysis revealed 7,523 DEGs in DDC-exposed mice vs. controls (*P* value < 0.05 and fold change > 1.5), with 1,002 DEGs identified between FZHY-treated and DDC groups (Fig. 3A-C). Intersectional analysis identified 703 overlapping DEGs, of which 550 exhibited counterregulatory expression patterns following FZHY intervention (Fig. 3A). Functional enrichment of DDC-upregulated/FZHY-downregulated genes highlighted predominant involvement in pro-inflammatory cascades, including cytokine-cytokine receptor interactions and NF-κB signaling (Fig. 3D). Conversely, DDC-downregulated/FZHY-upregulated genes were significantly enriched in the hepatic metabolism pathways, and based on *P*-value ranking, the most significantly enriched KEGG pathway was the PPAR signaling pathway (*P* = 3.33 × 10⁻⁶) (Fig. 3E). GSEA further validated these results: PPAR signaling demonstrated marked transcriptomic repression in DDC models (Fig. 3F), which was robustly reversed by FZHY (Fig. 3G).

**Fig. 4:**
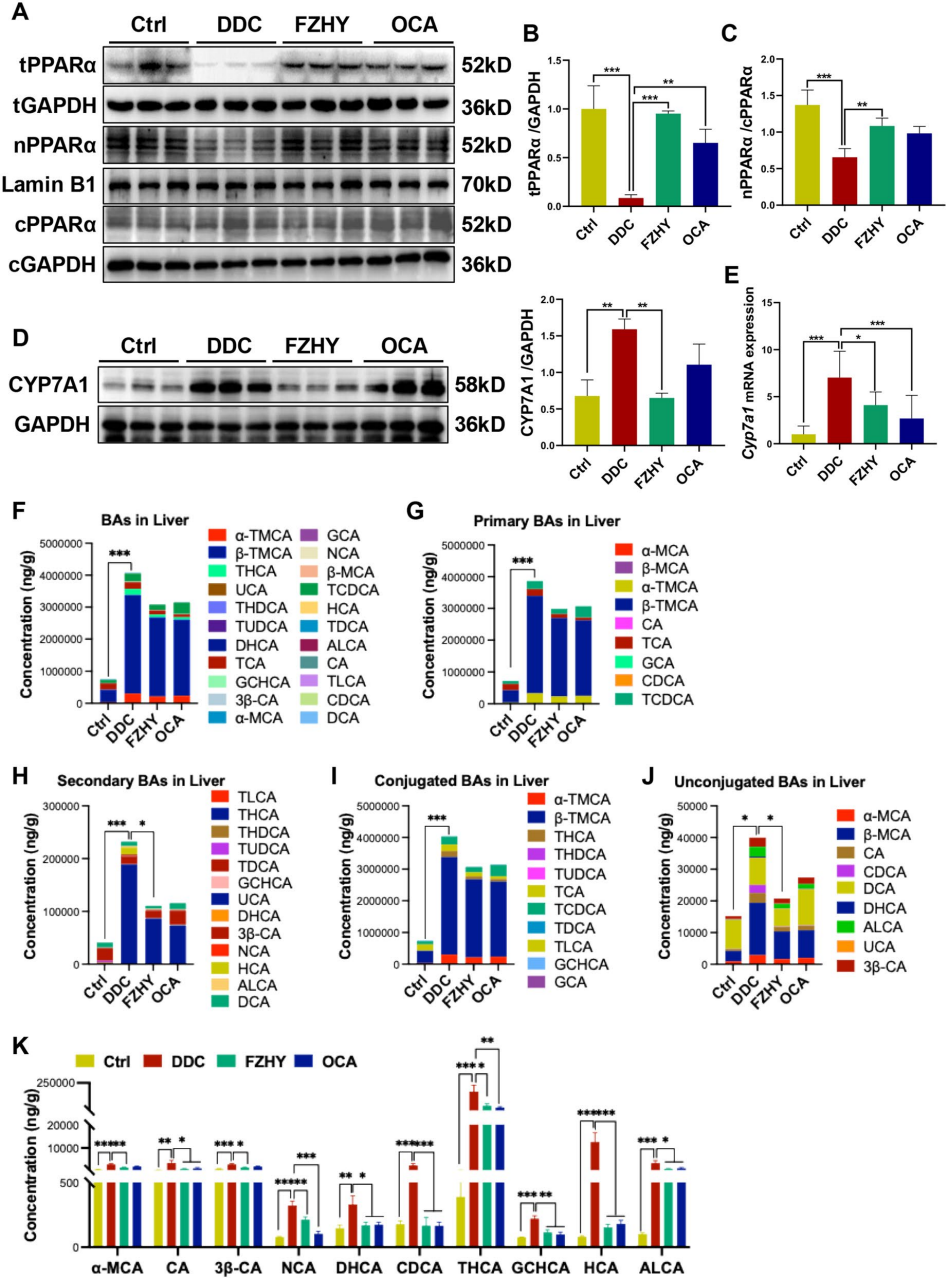
FZHY improved hepatic BAs metabolism by regulating PPARα signaling pathway. **A–C** FZHY intervention promoted the nuclear accumulation of PPARα. **D** immunoblotting and quantification of CYP7A1 and GAPDH served as the loading control. **E** *Cyp7a1* expression was determined by qRT-PCR. **F** Total BAs concentration. **G** Total primary BAs concentration. **H** Total secondary BAs concentration. **I** Total conjugated BAs concentration. **J** Total unconjugated BA concentration. **K** The concentrations of hepatic α-MCA, CA, 3β-CA, NCA, DHCA, CDCA, THCA, GCHCA, HCA and ALCA were detected. *P < 0.05; **P < 0.01; ***P < 0.001

Consistent with the transcriptomic data, our results showed that FZHY did not only enhance the total hepatic content of PPARα protein (*P* < 0.001), but also increased its nuclear to cytoplasmic ratio that was reduced by DDC inducing (*P* < 0.01) (Fig. 4A–C), indicating that FZHY could relieve chronic cholestatic liver injury symptoms by activating PPARα signaling. Moreover, FZHY significantly attenuated the DDC-driven overexpression of rate-limiting enzyme cholesterol 7a-hydroxylase (CYP7A1) (Fig. 4D, E). This coordinated suppression of CYP7A1 suggests FZHY might modulate the classical BAs synthesis pathway through PPARα-mediated transcriptional regulation.

Quantitative LC–MS/MS analysis revealed significant increases in the total hepatic BAs levels, and primary and secondary BAs levels following DDC exposure (*P* < 0.001), FZHY intervention effectively normalized BAs homeostasis, achieving reversal of pathologically elevated BAs levels, especially significantly reduced the secondary BAs levels (*P* < 0.05) (Fig. 4F, G). Furthermore, we determined the conjugated and unconjugated BAs in mice. DDC-induced mice showed higher levels of conjugated and unconjugated BAs (*P* < 0.001, *P* < 0.05) (Fig. 4I, J), while FZHY significantly reduced the hepatic unconjugated BAs levels (*P* < 0.05) (Fig. 4J). In addition, the most pronounced therapeutic effects were observed for α-muricholic acid (α-MCA), cholic acid (CA), 3β-cholic acid (3β-CA), norcholic acid (NCA), dihydroxycholestanoic acid (DHCA), chenodeoxycholic acid (CDCA), accompanied by trihydroxycholestanoic acid (THCA), glycochenodeoxycholic acid (GCHCA), hyocholic acid (HCA) and allocholic acid (ALCA) (*P* < 0.05, *P* < 0.01) (Fig. 4K).

### FZHY reduced hepatic inflammation in DDC-induced chronic cholestatic liver injury mice

**Fig. 5:**
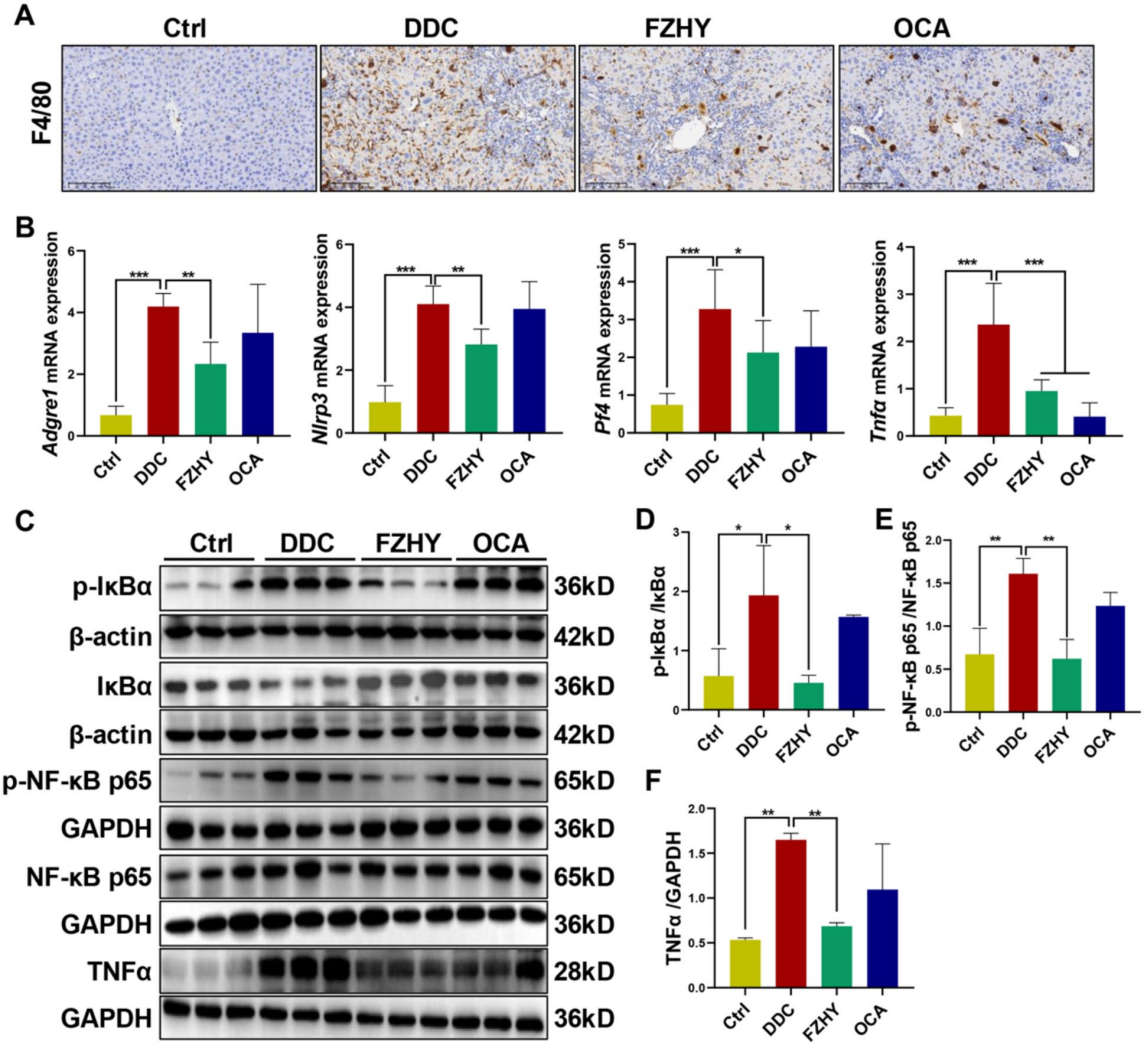
FZHY reduced hepatic inflammation in DDC-induced mice. **A** liver sections were immunohistochemistry stained with F4/80 (scale bar = 100 µm). **B** *Adgre4*, *Nlrp3*, *Pf4* and *Tnfα* expressions were determined by qRT-PCR. **C–F** immunoblotting and quantification of p-IκBα, IκBα, p-NF-κB p65, NF-κB p65, Tnfα, β-actin and GAPDH served as the loading control. *P < 0.05; **P < 0.01; ***P < 0.001

DDC-fed mice had a more severe inflammatory response, so we looked into whether FZHY treatment could help with inflammation as well. There was an increase in F4/80 positive macrophages, as well as hepatic mRNA expression of *Adgre1*, *Nlrp3*, *Pf4* and *Tnfα* in DDC-induced mice compared with control mice, which were reversed by FZHY (*P* < 0.05, *P* < 0.01, *P* < 0.001) (Fig. 5A, B). The nuclear factor kappa-B (NF-κB) is an important mediator of inflammatory responses and contributes to the regulation of immune homeostasis [14]. In this study, DDC exposure activated canonical NF-κB signaling, evidenced by increased phosphorylation of inhibitor of NF-κB α (IκBα) and NF-κB p65 (expressed as a ratio of p-IκBα and IκBα, p-NF-κB p65/NF-κB p65) (*P* < 0.01), FZHY intervention suppressed these activation states respectively (*P* < 0.01) (Fig. 5C–E), corroborating our earlier RNA-seq findings (Fig. 3D). In line with qRT-PCR results, the hepatic protein expression of TNFα was significantly upregulated induced by DDC (*P* < 0.01), which was remarkably reduced in FZHY-treated mice compared with the DDC-induced mice (*P* < 0.01) (Fig. 5C, F).

### FZHY failed to ameliorated BAs accumulation and hepatic inflammation induced by DDC in *Pparα*^−/−^ mice

**Fig. 6:**
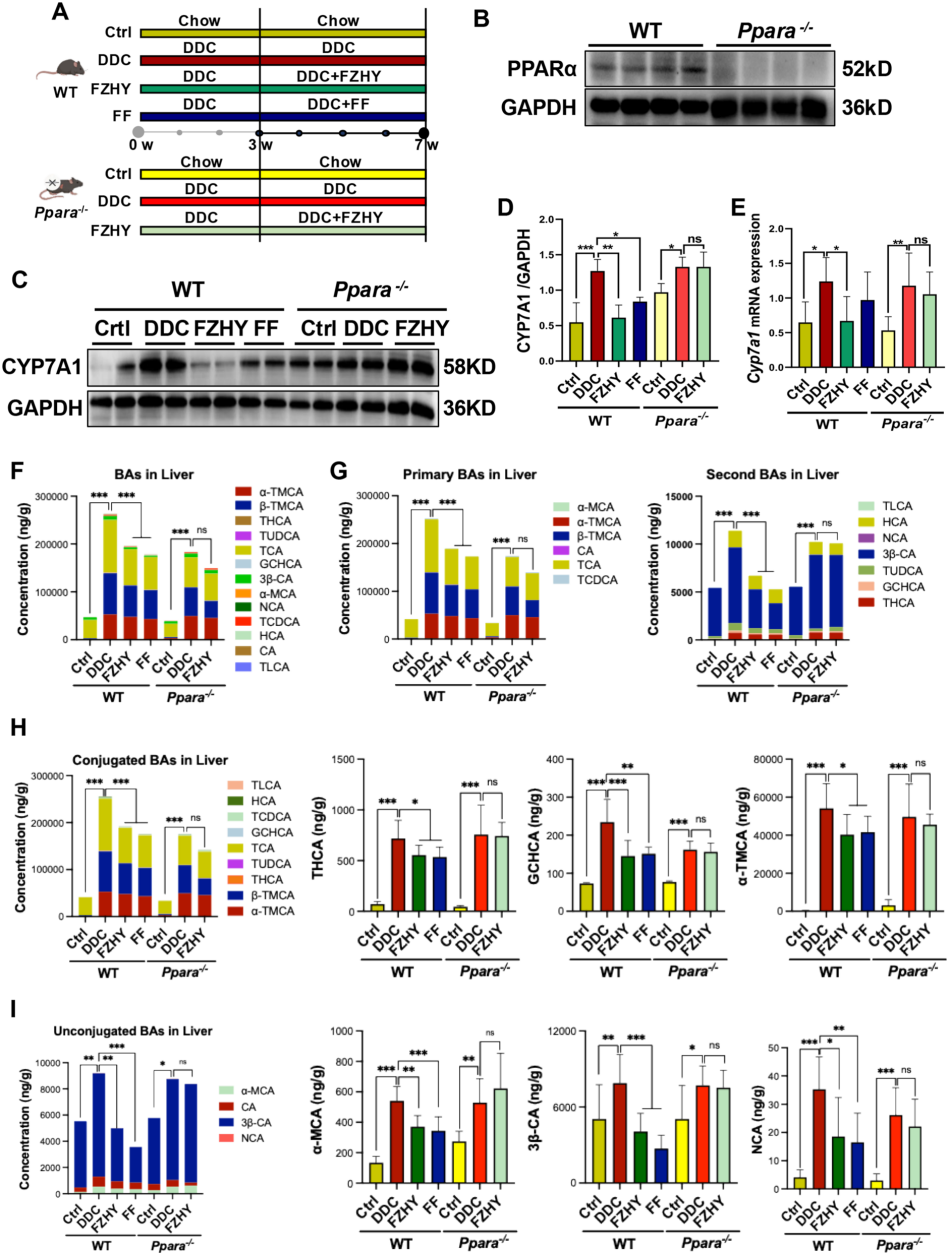
Genetic ablation of *Pparα* abrogated FZHY’s regulating DDC-induced bile acids metabolism. **A** Schematic diagram illustrating the experimental design of DDC-induced mice treated with FZHY in WT mice and *Pparα*^−/−^ mice. **B–D** Immunoblotting and quantification of CYP7A1 and GAPDH served as the loading control. **E** *Cyp7a1* expression were determined by qRT-PCR. **F** Total major BAs concentration. **G** Total major primary and secondary BAs concentration. **H** Total and individual major conjugated BAs concentration. **I** Total and individual major unconjugated BA concentration. *P < 0.05; **P < 0.01; ***P < 0.001

To elucidate whether FZHY protects against chronic cholestatic liver injury in a manner dependent on PPARα, both age and sex matched WT mice and *Pparα*^−/−^ mice were fed with DDC diet for 7 weeks and treated with or without FZHY, and fenofibrate was used as a positive control (Fig. 6A). Fenofibrate has been investigated both in the laboratory and human pilot studies as second-line therapy in combination with UDCA and was found to significantly reduce serum markers of liver injury and the toxicity of the total BAs pool for patients with PBC [15] and also for those with PSC who experience an insufficient response to UDCA therapy [16]. The western blot analysis confirmed complete ablation of PPARα protein expression in *Pparα*^−/−^ mice, while maintaining physiological expression levels in WT livers (Fig. 6B). As expected, the hepatic protein and gene expressions of CYP7A1 were significantly upregulated induced by DDC in WT mice (*P* < 0.05, *P* < 0.001), which was remarkably reduced in both FZHY-treated and fenofibrate-treated mice compared with the DDC-induced mice (*P* < 0.05, *P* < 0.01) (Fig. 6C–E). While, the beneficial effect of FZHY treatment on CYP7A1 was abolished by *Pparα* gene knockout (Fig. 6C–E). Accompanyingly, the absence of PPARα essentially abrogated the ameliorative effect of FZHY on BAs accumulation, as reflected by the total major BAs concentration (Fig. 6F), total major primary and secondary BAs concentration (Fig. 6G), total major conjugated and unconjugated BAs concentration, as well as hepatic THCA, GCHCA, α-TMCA, α-MCA, 3β-CA and NCA levels (Fig. 6H, I).

**Fig. 7:**
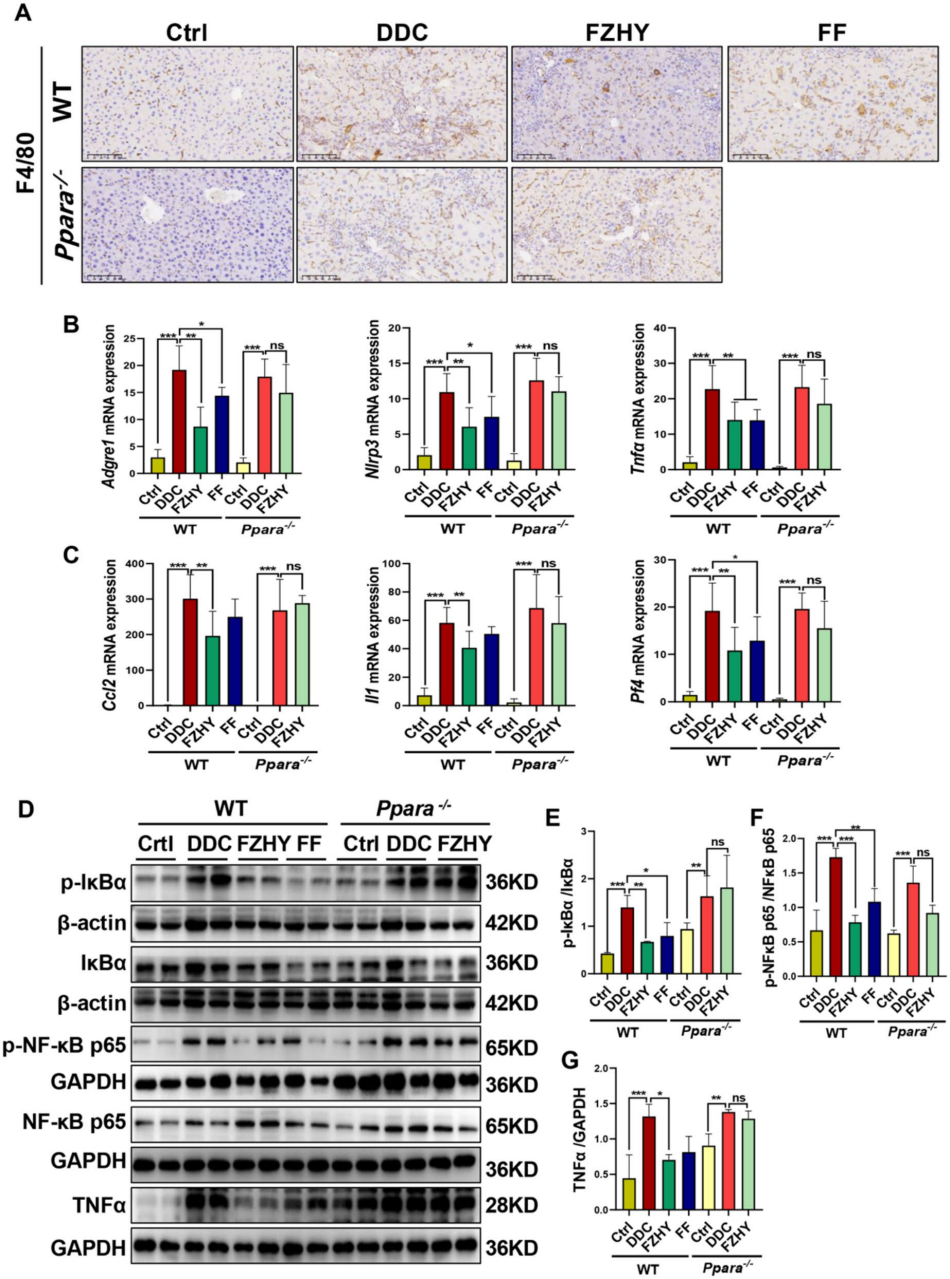
Genetic ablation of *Pparα* abrogated FZHY’s therapeutic efficacy against DDC-induced hepatic inflammation. **A** Liver sections were immunohistochemistry stained with F4/80 (scale bar = 100 µm). **B, C** *Adgre4*, *Nlrp3*, *Tnfα*, *Ccl2*, *Il1* and *Pf4* expressions were determined by qRT-PCR. **D–G** Immunoblotting and quantification of p-IκBα, IκBα, p-NF-κB p65, NF-κB p65, Tnfα, β-actin and GAPDH served as the loading control. *P < 0.05; **P < 0.01; ***P < 0.001

Moreover, FZHY reversed the increase in F4/80 positive macrophages, hepatic mRNA expressions of *Adgre1*, *Nlrp3*, *Pf4* and *Tnfα*, and the phosphorylation of IκBα and NF-κB p65 induced by DDC in WT mice (*P* < 0.05, *P* < 0.01, *P* < 0.001) (Fig. 7), while there was no significant difference between the FZHY-treated group and DDC-induced group in *Pparα*^−/−^ mice (Fig. 7), suggesting the regulatory effects of FZHY on the inflammatory response were eradicated due to the absence of PPARα.

### FZHY ameliorated ductular reaction and biliary fibrosis induced by DDC in mice, which depended on PPARα

**Fig. 8:**
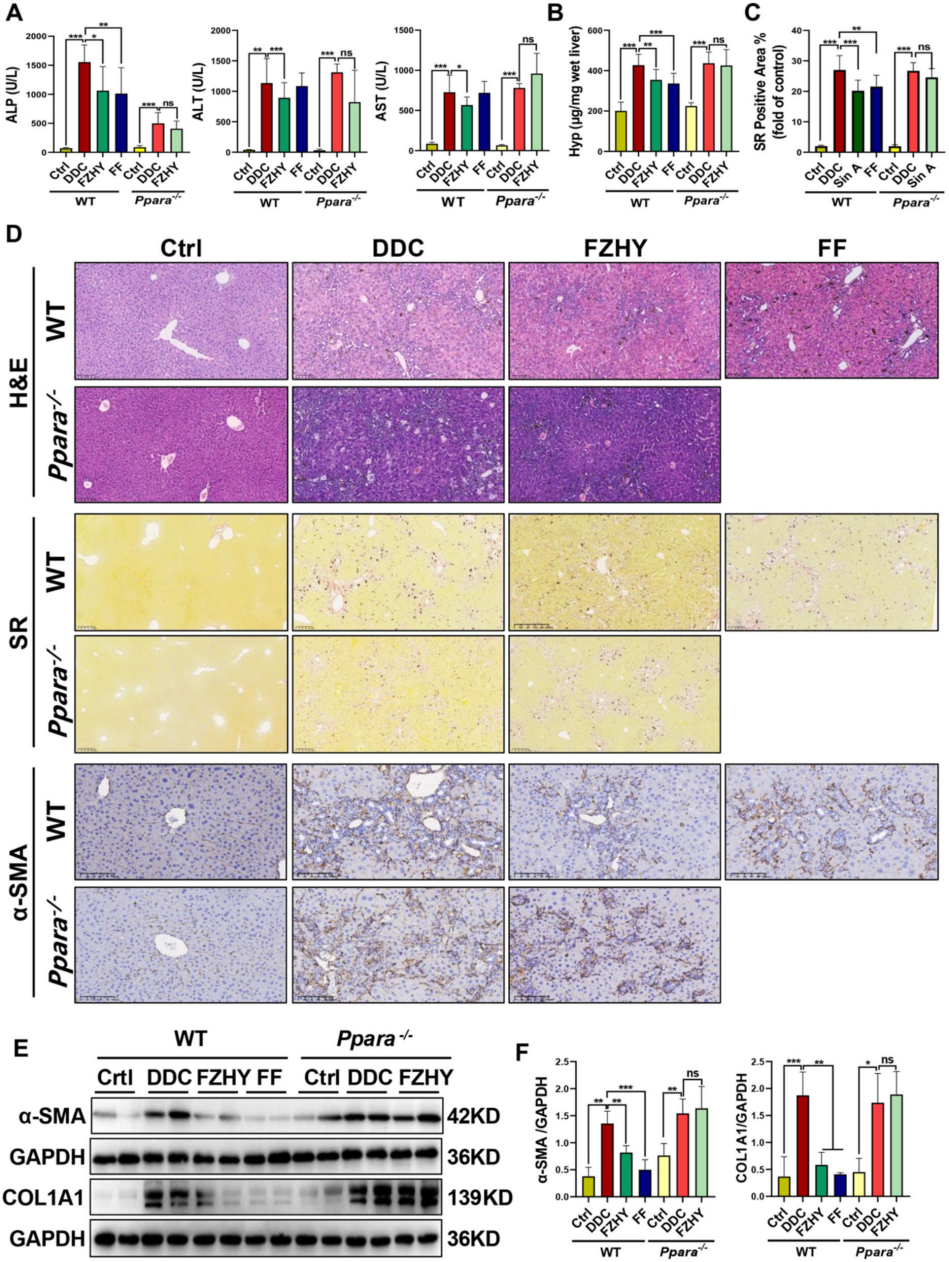
Genetic ablation of *Pparα* abrogated FZHY’s therapeutic efficacy against DDC-induced liver fibrosis. **A** Serum levels of ALP, ALT and AST. **B** Hepatic collagen content was biochemically determined by Hyp analysis. **C** Collagen morphometry (%) of SR^+^ area. **D** Liver sections were stained with H&E (scale bar = 100 µm), SR staining (scale bar = 200 µm) and α-SMA (scale bar = 100 µm). **E, F** Immunoblotting and quantification of COL1A1 and α-SMA. *P < 0.05; **P < 0.01; ***P < 0.001

Quantitative analysis revealed FZHY significantly attenuated DDC-induced hepatobiliary injury biomarkers in WT mice, ALP (*P* < 0.05), ALT (*P* < 0.001) and AST (*P* < 0.05) levels, but this therapeutic efficacy was abrogated in *Pparα*^−/−^ mice (*P* > 0.05) (Fig. 8A). SR morphometry showed significant reduction in collagen deposition area in WT-FZHY group vs. WT-DDC group (*P* < 0.001), while *Pparα*^−/−^ cohorts showed no significant improvement (Fig. 8C, D). Hepatic Hyp content, hepatic mRNA and protein expressions of COL1A1 and a-SMA paralleled these findings, with the remarkably reduction in DDC-induced mice caused by FZHY ceased due to PPARα deficiency (Fig. 8B, D–F).

**Fig. 9:**
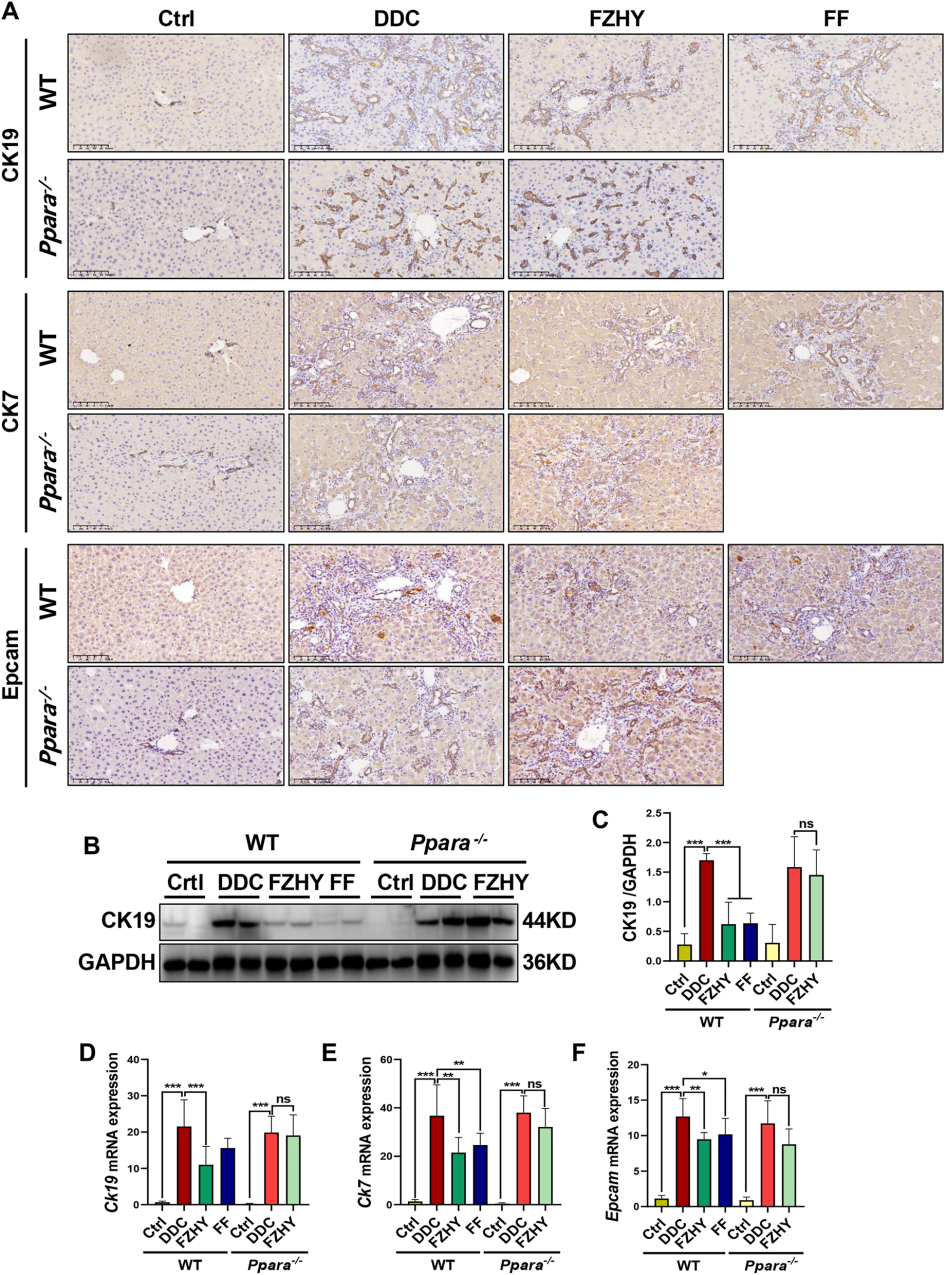
Genetic ablation of *Pparα* abrogated FZHY’s therapeutic efficacy against DDC-induced ductular reaction. **A** Liver sections were stained with CK19 (scale bar = 100 µm), CK7 (scale bar = 100 µm) and Epcam (scale bar = 100 µm). Representative images are shown. **B, C** Immunoblotting and quantification of CK19. **D–F** *Ck19*, *Ck7* and *Epcam* expressions were determined by RT-qPCR. *P < 0.05; **P < 0.01; ***P < 0.001

Consistent with mechanistic predictions, genetic ablation of *Pparα* abrogated FZHY’s therapeutic efficacy against DDC-induced ductular reaction, as evidenced by immunohistochemical saining of CK19, CK7 and Epcam (Fig. 9A), the western blot result of CK19 (Fig. 9B) and the qRT-PCR results of *Ck19*, *Ck7* and *Epcam* (Fig. 9D–F). These findings collectively indicate that the effectiveness of FZHY in chronic cholestatic liver injury mice is abolished by PPARα deficiency; this suggests that the therapeutic effects of FZHY on chronic cholestatic liver injury is dependent on PPARα.

## Discussion

FZHY, comprising *Radix Salvia miltiorrhiza* (Danshen), *Persicae semen* (Taoren), *Cordyceps* (Dongchongxiacao), *Gynostemma pentaphylla* (Jiaogulan), *Schisandrae chinensisfructus* (Wuweizi) and *Pini pollen* (Songhuafen), was approved by Chinese State Food and Drug Administration (NO: Z20050546) in 2002 for the treatment of liver fibrosis in China. It represents a promising therapeutic option, demonstrating efficacy in delaying and reversing liver fibrosis while concurrently ameliorating patient clinical symptoms [17]. However, the impact of FZHY on cholestatic liver disease has not been fully elucidated. To investigate the effects of FZHY on chronic cholestatic liver injury, we conducted experiments on DDC-induced mice. Our objective was to determine whether FZHY exerts hepatoprotective effects against chronic cholestatic liver injury comparable to both OCA and fenofibrate, thereby identifying a potential clinically applicable alternative for patients.

During cholestasis, elevated levels of BAs accumulate in both serum and liver [18], leading to hepatic injury. Impairments in adaptive responses to elevated BAs concentrations result in intrahepatic BAs retention and BA-mediated hepatotoxicity. This pathological cascade can progress to fibrosis, cirrhosis, and ultimately hepatocellular carcinoma [19]. The maintenance of BAs metabolic homeostasis is intrinsically linked to the pathogenesis of cholestatic liver diseases and cholangiopathies [20]. Therefore, therapeutic modulation of BAs metabolism represents a beneficial strategy for mitigating cholestatic liver injury [21]. BAs are synthesized from cholesterol via two major pathways: the classical pathway, which is liver-specific, and the alternative pathway, which can occur in both the liver and extrahepatic tissues. The rate-limiting enzyme of the classical pathway, CYP7A1, primarily generates CA and CDCA [22]. Notably, CDCA and its glycine conjugate, GCDCA, are directly cytotoxic to hepatocytes, promoting cell death. CA, in turn, acts as a potent pro-inflammatory mediator and activator of hepatic stellate cells, thereby driving fibrogenesis. Furthermore, the hepatic accumulation of synthesis precursors such as THCA and DHCA reflects a dysfunctional and obstructed BAs biosynthetic pathway. Concomitant reductions in secondary BAs like α-MCA and HCA further suggest a potential modulation of the gut-liver axis [23].

In our study, FZHY treatment significantly attenuated the DDC-induced increase in total hepatic BAs levels in mice, with a pronounced decrease in the content of CA and CDCA. This effect may be attributed to a marked upregulation of CYP7A1 expression. Importantly, the reduction by FZHY of a broad spectrum of BAs, including CDCA, GCDCA, CA, α-MCA, HCA, 3β-CA, NCA, THCA, DHCA, and ALCA, indicates that its therapeutic action extends beyond merely alleviating the pathological BAs overload. Rather, FZHY appears to enhance the overall efficiency of hepatic BAs synthesis and promote metabolic turnover, thereby restoring BAs homeostasis.

In addition to the direct damage caused by BAs, inflammation is an important cause of cholestasis. Moreover, BAs is the main trigger of inflammation in cholestatic liver injury [24]. Cholestatic liver injury is accompanied by significant inflammatory response, characterized by a large number of inflammatory cell infiltration and activation of inflammatory pathways. Dysregulated NF-κB signaling pathway is involved in the pathogenesis of various inflammation-related diseases and is considered a promising therapeutic target [25]. The abovementioned results revealed that the NF-κB signaling pathway was activated during the progression of cholestatic liver injury by DDC. FZHY treatment in this study suppressed the NF-κB signaling pathway and decreased the infiltration levels of F4/80 positive macrophages in the liver and inflammatory factors, NLRP3, PF4 and TNFα. Therefore, oral administration of FZHY resulted in a significant ameliorating BAs accumulation and hepatic inflammation.

It is well known that cholestatic injury can cause massive bile duct hyperplasia in the portal area, a typical pathological manifestation of chronic cholestatic liver diseases, and subsequently causes ductular reaction and liver fibrosis [26, 27]. Our study demonstrated that FZHY mitigated the DDC-increased hepatic Epcam, CK7 and CK19, and also ameliorated biliary fibrosis in DDC-induced mice by decreasing the SR-positive area, hepatic Hyp content and the expression of α-SMA and COL1A1. These results provide evidence that FZHY significantly ameliorated chronic cholestatic liver injury phenotypes in mice.

RNA-seq analysis was further employed to elucidate the molecular mechanism underlying FZHY’s efficacy against chronic cholestatic liver injury. The results demonstrated that the effect of FZHY on chronic cholestatic liver injury was closely associated with activiating the PPARα signaling pathway and suppressing the NF-κB signaling. PPARα exhibits predominant expression in tissues characterized by high rates of fatty acid oxidation, including the liver, heart, adipose tissue, and kidneys [28]. Due to its regulatory role in the multi-enzymatic processes governing BAs synthesis and metabolism, particularly through the inhibition of CYP enzymes such as CYP7A1, PPARα represents a promising pharmacological target for cholestatic liver diseases. PPARα activators are well-established suppressors of *Cyp7a1* gene promoter activity in human hepatoma HepG2 cells [29, 30], and PPARα agonists downregulate *Cyp7a1* and *Cyp27a1* expression and activity in rodents [31]. In the present study, FZHY not only significantly increased total hepatic PPARα protein levels but also restored its nuclear-to-cytoplasmic ratio, which was diminished by DDC challenge. Meanwhile, the level of CYP7A1, which encode the classic rate-limiting enzymes for BAs synthesis, was significantly decreased after treatment with FZHY. To determine the necessity of PPARα activation for FZHY’s anti-cholestatic effects, experiments were conducted utilizing both WT and *Ppara*^−/−^ mice, with the selective PPARα agonist fenofibrate serving as the positive control. Our findings reveal that genetic ablation of PPARα abolished FZHY-induced downregulation of CYP7A1 and attenuated its reduction of BAs accumulation.

Furthermore, activated PPARα exerts anti-inflammatory effects through transrepression of pro-inflammatory transcription factors [32]. Specifically, PPARα activation suppresses IκBα expression in human primary hepatocytes, resulting in the retention of NF-κB in the cytosol and inhibition of its nuclear translocation and DNA binding, thereby attenuating inflammatory responses. Consistent with this mechanism, FZHY treatment inhibited NF-κB signaling activation and suppressed inflammatory cytokine secretion in WT mice, which is similar to the therapeutic effect of fenofibrate. However, this anti-inflammatory efficacy of FZHY was abolished in *Ppara*^−/−^ mice. Critically, genetic ablation of *Ppara* also reversed FZHY’s therapeutic benefits against cholestasis-associated liver injury, ductular reaction, and hepatic fibrosis as observed through assessment of serum markers of liver injury and cholangiocyte markers, SR staining analysis and quantification of hepatic Hyp. Interestingly, genetic ablation of *Ppara* did not significantly exacerbate chronic cholestatic liver injury phenotypes in DDC-induced mice, including BAs accumulation, hepatic inflammation, ductular reaction and liver fibrosis, which was consistent with the results of other published studies [33]. This clearly demonstrates that FZHY alleviates chronic cholestatic liver injury in DDC-induced mice in a PPARα-dependent manner. Our study also had some limitations. While we demonstrate that FZHY alleviates chronic cholestatic liver injury by upregulating PPARα, the mechanism underlying this upregulation, whether mediated through transcriptional activation, protein stabilization, or modulation of upstream signaling pathways, remains unclear and warrants further investigation. Furthermore, since FZHY is a complex mixture, investigating its active monomeric components that upregulate PPARα represents a promising direction for future research.

## Conclusion

The present study underscores FZHY alleviated BAs accumulation and hepatic inflammation by upregulating PPARα, and subsequently ameliorated ductular reaction and liver fibrosis in DDC-induced mice. The findings not only establish PPARα activation as a novel pharmacological mechanism underlying FZHY’s therapeutic effects but also provide experimental evidence supporting FZHY’s potential as a promising therapeutic agent for chronic cholestatic liver injury.

## Supplementary Information

Additional file 1.

## Data Availability

All relevant data are available from the corresponding authors upon reasonable request. RNA-Seq data will be uploaded to GenBank for public access when the manuscript is published.

## Abbreviations

α-MCA: α-Muricholic acid
α-TMCA: α-Tauromuricholic acid
3β-CA: 3β-Cholic acid
ALCA: Allocholic acid
ALT: Alanine aminotransferase
AST: Aspartate aminotransferase
BAs: Bile acids
CA: Cholic acid
CCl₄: Carbon tetrachloride
CDCA: Chenodeoxycholic acid
CYP7A1: Cholesterol 7a-hydroxylase
DDC: 3,5-Diethoxycarbonyl-1,4-dihydrocollidine
DEGs: Differentially expressed genes
DHCA: Dihydroxycholestanoic acid
FBS: Fetal bovine serum
FF: Fenofibrate
FZHY: Fuzheng Huayu formula
GCHCA: Glycochenodeoxycholic acid
GSEA: Gene set enrichment analysis
HCA: Hyocholic acid
H&E: Hematoxylin and eosin
HSCs: Hepatic stellate cells
Hyp: Hydroxyproline
NCA: Norcholic acid
NF-κB: Nuclear factor kappa-B
OCA: Obeticholic acid
PBC: Primary biliary cholangitis
PPARα: Peroxisome proliferator-activated receptor a
PSC: Primary sclerosing cholangitis
qRT-PCR: Quantitative real-time polymerase chain reaction
IκBα: Inhibitor of NF-κB α
SR: Sirius red
TDCA: Taurodeoxycholic acid
THCA: Trihydroxycholestanoic acid
WT: Wild-type
