# Correction: Fuzheng Huayu formula ameliorates chronic cholestatic liver injury by upregulating PPARa in mice

**DOI:** 10.1186/s13020-026-01419-8

**Published:** 2026-05-14

**Authors:** Zheng Zhang, En-qi Tang, Chun-hui Li, Bi-bi Wang, Yue Liang, Jin-xin Lv, Gao-feng Chen, Wei Liu, Yong-ping Mu, Ping Liu, Jia-mei Chen

**Affiliations:** 1 Institute of Liver Diseases, Key Laboratory of Liver and Kidney Diseases (Ministry of Education), Shuguang Hospital Affiliated to Shanghai University of Traditional Chinese Medicine, 528 Zhangheng Road, Shanghai, 201203 China; 2 Institute of Interdisciplinary Medicine, Shanghai University of Traditional Chinese Medicine, Shanghai, 201203 China

**Correction** **:** **Chinese Medicine (2026) 21:93**

10.1186/s13020-026-01368-2

Following publication of the original article [1], the authors identified an unintentional image placement error in Fig. 9A, which presents the CK19 immunohistochemistry results. During data organization and figure preparation, an oversight in adjusting the vertical positioning of the images led to the image intended for the *Pparα*^*−/−*^ control group being inadvertently replaced with that of the WT control group. This error occurred purely by accident during figure assembly. The correct Fig. 9 has been provided in this Correction.

The incorrect Fig. 9 is:

**Fig. 9:**
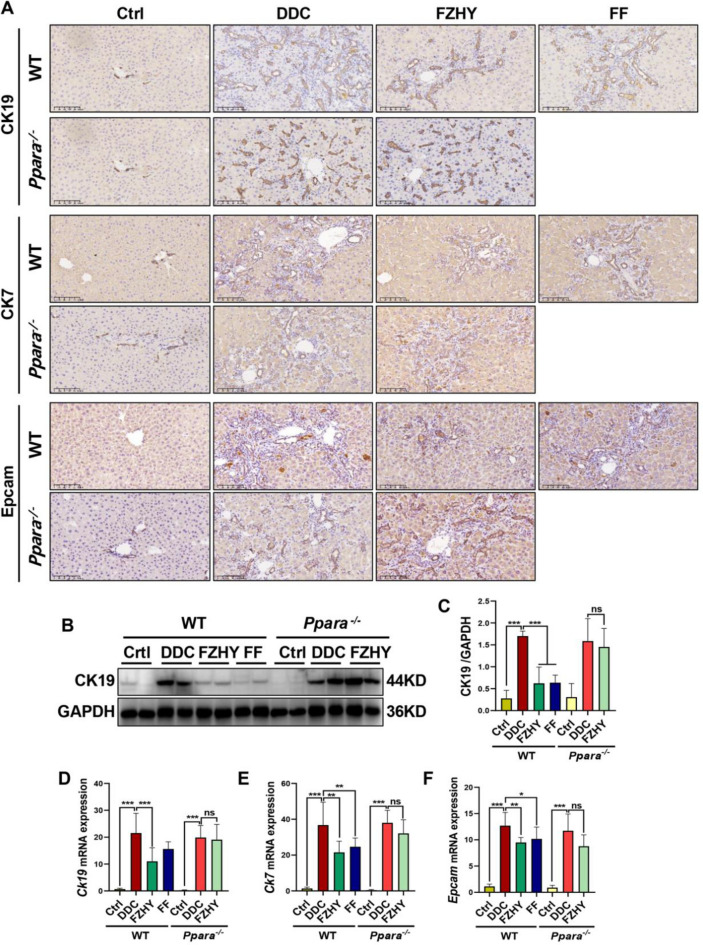
Genetic ablation of Pparα abrogated FZHY’s therapeutic efficacy against DDC-induced ductular reaction. **A** Liver sections were stained with CK19 (scale bar = 100 µm), CK7 (scale bar = 100 µm) and Epcam (scale bar = 100 µm). Representative images are shown. **B**, **C** Immunoblotting and quantification of CK19. **D–F** *Ck19*, *Ck7* and *Epcam* expressions were determined by RT-qPCR. *P < 0.05; **P < 0.01; ***P < 0.001

The correct Fig. 9 is:

**Fig. 9:**
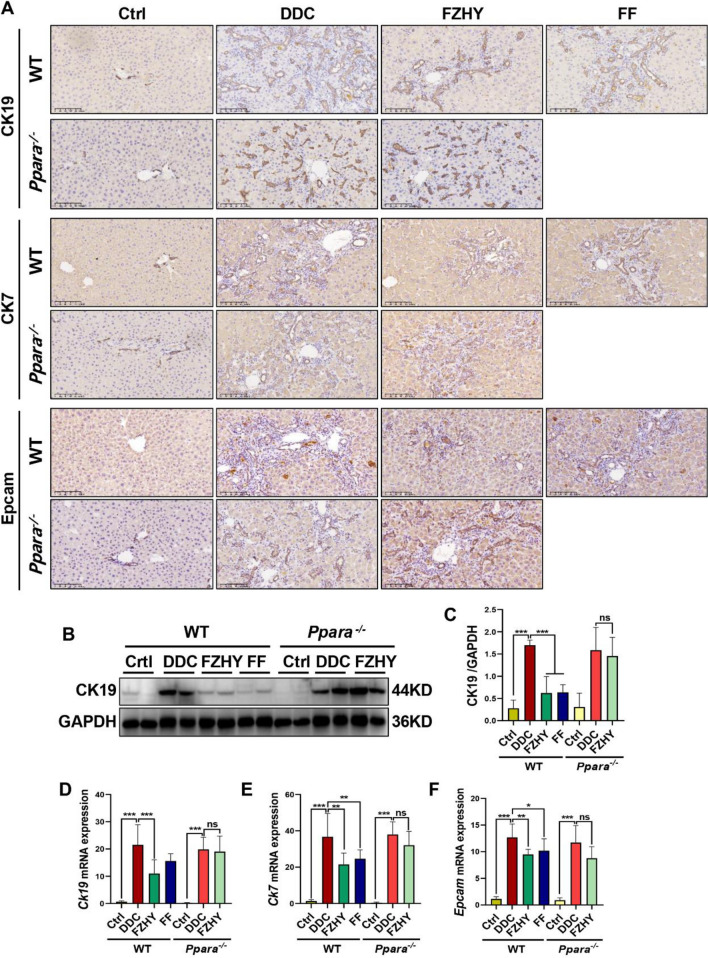
Genetic ablation of Pparα abrogated FZHY’s therapeutic efficacy against DDC-induced ductular reaction. **A** Liver sections were stained with CK19 (scale bar = 100 µm), CK7 (scale bar = 100 µm) and Epcam (scale bar = 100 µm). Representative images are shown. **B**, **C** Immunoblotting and quantification of CK19. **D–F** *Ck19*, *Ck7* and *Epcam* expressions were determined by RT-qPCR. *P < 0.05; **P < 0.01; ***P < 0.001

The original article [1] has been corrected.
