# Supplementary material for: Fuzheng Huayu formula ameliorates chronic cholestatic liver injury by upregulating PPARa in mice
Source: Chin Med. 2026 Mar 18;21:93. doi: 10.1186/s13020-026-01368-2 (PMC12998289; doi:10.1186/s13020-026-01368-2)
Supplement: Supplementary file 1 — Additional file 1. [file 13020_2026_1368_MOESM1_ESM.docx]

**Supplementary Tables**

**Supplementary Table 1. Antibodies information used in this Study. IHC, immunohistochemistry; WB, western blot.**

| **Application** | **Specificity** | **Catalog#** | **Vendor** | **Dilution** |
| --- | --- | --- | --- | --- |
| IHC | α-SMA | ab124964 | Abcam | 1:1000 |
| IHC | F4/80 | 70076 | CST | 1:250 |
| IHC | Col-I | ab34710 | Abcam | 1:200 |
| IHC | CK19 | 10712-1-AP | Proteintech | 1:200 |
| IHC | CK7 | 15339-1-AP | Proteintech | 1:200 |
| IHC | Epcam | Ab213500 | Abcam | 1:200 |
| WB | α-SMA | ab5694 | Abcam | 1:500 |
| WB | CK19 | 10712-1-AP | Proteintech | 1:1000 |
| WB | TNFα | 11948 | CST | 1:1000 |
| WB | p-NF-κB p65 | 3033 | CST | 1:1000 |
| WB | NF-κB p65 | 8242 | CST | 1:1000 |
| WB | CYP7A1 | ab65596 | Abcam | 1:1000 |
| WB | PPARα | ab215270 | Abcam | 1:2000 |
| WB | β-actin | ab71916 | Proteintech | 1:10000 |
| WB | Lamin B1 | 66009-1-Ig | Abcam | 1:1000 |
| WB | GAPDH | 60004-1-Ig | Proteintech | 1:10000 |
| WB | HRP-labeled Goat Anti-Rabbit IgG | A0208 | Beyotime | 1:1000 |
| WB | HRP-labeled Goat Anti-Mouse IgG | A0206 | Beyotime | 1:1000 |

**Supplementary Table 2. Primer sequences of qRT-PCR used in this Study.**

| Gene | Forward primer (5’ to 3’) | Reverse primer (5’ to 3’) |
| --- | --- | --- |
| *Acta2* | AGACCTTCAATGTCCCTGCCA | GTTGTGAGTCACGCCATCTCC |
| *Col1a1* | TGTCTGGTTTGGAGAGAGCA | AGTGATAGGTGATGTTCTGG |
| *Ck19* | CAGGTCGCTGTCCACACTA | TATCTCTGCCACAGTGCCTT |
| *Ck7* | CGGAATGAGATTGCGGAGAT | CCTTGTTCCTCAGCCTCTGC |
| *Epcam* | GGCGTGGAACTCAGAACTTA | TCTACTGTGGGCTGTTTATG |
| *Cyp7a1* | GTGATGTTTGAAGCCGGATATC | TTTATGTGCGGTCTTGAACAAG |
| *Nlrp3* | GCCTTGAAGAAGAGTGGATGC | CTGCGTGTAGCGACTGTTG |
| *Tlr4* | GAGCCGGAAGGTTGTGGTAGTG | AGGACAATGATGATGCCAGAGC |
| *Il1* | GAAATGCCACCTTTTGACAGTG | TGGATGCTCTCATCAGGACAG |
| *Ccl2* | CATCTGCCCTAAGGTCTTCA | GAGGTGGTTGTGGAAAAGGTA |
| *Adgre1* | CGTGTTGTTGGTGGCACTGTGA | CCACATCAGTGTTCCAGGAGAC |
| *Tnfα* | CCCAATCTGTGTCCTTCTAA | CACTACTTCAGCGTCTCGTG |
| *Ppara* | GAGGATGGGGACTTTTGTTCT | GGCTTTTTGGCTGTAGGAGG |
| *Gapdh* | CATCACTGCCACCCAGAAGACTG | ATGCCAGTGAGCTTCCCGTTCAG |
